# Psychometric evaluation of the German version of the patient activation measure (PAM13)

**DOI:** 10.1186/1471-2458-13-1027

**Published:** 2013-10-30

**Authors:** Jördis M Zill, Sarah Dwinger, Levente Kriston, Anja Rohenkohl, Martin Härter, Jörg Dirmaier

**Affiliations:** 1 Department of Medical Psychology, University Medical Center Hamburg-Eppendorf, Martinistr. 52, 20246 Hamburg, Germany

## Abstract

**Background:**

The Patient Activation Measure (PAM) consists of 13 items and assesses patient (or consumer) self-reported knowledge, skills, and confidence for self-management of one’s health or chronic condition. The aim of this study was to translate the original American version of the PAM13 into German and to test the psychometric properties of the German version in an elderly, multimorbid population with various chronic conditions.

**Methods:**

Translation was performed by a standardized forward-backward translation process. The PAM13 was sent to 9.075 participants enrolled in a randomized controlled study. 4.306 participants responded to the questionnaire. Descriptive and reliability analyses were carried out. To examine scale properties, Andrich’s Rasch Rating Scale Model was fitted.

**Results:**

The internal consistency is good (α = 0.88) and the item-rest-correlations were found as strong to moderate. The unidimensionality of the construct was confirmed, with a variance explanation of 40.9% and good model-fits for the Rasch model. However, the lowest response options were very rarely used across all items (below 5%) and ranking order of items according to their difficulty was substantially different from that of the American version. Differential item functioning (DIF) was found in subgroups (sex, age, health status), but differences were small.

**Conclusion:**

The German version of the PAM13 showed acceptable reliability and the model-fit statistics confirmed the Rasch model. The different ranking order of the items and the unfair distribution of the response options suggest further research on validation and revision of the construct.

## Background

Increasing ageing, longer life expectancy and advances in health care lead to a greater number of people living with chronic illnesses and growing demands on the health care systems [1,2]. As technological progress alone cannot compete with the resulting challenges for health care systems, there are various efforts to improve the quality of chronic illness care. One focus of these efforts has recently been put on the patient activation for self-management one’s diseases [3]. Activation of patients, referring to the confidence that enables patients to become actively engaged in their health, is central within new models of chronic illness care, like for example the framework of the Chronic Care Model [4,5]. In this framework, improvement of self-management strategies strengthens the patients’ activation levels in the course of the treatment in addition to other components (e.g. proactive team interaction). Self-management of chronic diseases refers to aspects like medication use, life-style changes, and health behavior changes to prevent long-term complications or increase adherence to treatment regimens.

Current findings show that the degree of patients’ activation differs significantly for those with a low activation level having higher costs than patients with a high activation level [6]. Additionally, research shows that if patients are more engaged and play a more active role in their health care is associated with greater satisfaction with care, increased adherence, more knowledge, improved health status and a decreased utilization of health care services [7].

However, especially older patients with various diseases have problems participating actively in their treatment. Success of self-management is likely to depend on self-management skills and patient activation [8]. Therefore, it is important to identify patients lacking these skills, to develop and evaluate interventions to enhance the skills necessary for activation and to tailor support for self-management by adjusting the intervention to the individual patients’ level of activation. To attain these goals, it is crucial to have valid and reliable assessment methods for the individual level of activation.

### Patient activation

In 2004, Hibbard et al. [9] developed a model of patient activation supposed to inform about strategies to increase patient engagement in health. Patient activation is defined as the degree to which a patient knows that one must engage actively in managing his disease, its consequences and the corresponding health care, and the degree one feels able to meet the challenges of that patients’ role [10]. Expert consensus and patient focus groups [11] identified four relevant elements for coping with chronic diseases: knowledge, skills, confidence and behaviors critical for coping with chronic diseases. Hibbard and colleagues [9] conceptualized patient activation as a hierarchically structured developmental process with four stages of activation, where individuals can progress through to become fully activated in their health management. (1) Believes Active Role Important (2) Confidence and Knowledge to Take Action (3) Taking Action (4) Staying the Course under Stress.

### The patient activation measure (PAM)

Hibbard and colleagues developed the Patient Activation Measure (PAM) on this above mentioned concept of patient activation. The PAM was originally developed as a 22-item version using qualitative methods, classical test theory, and Rasch analysis [10]. The result is a unidimensional, interval-level, Guttman-type scale [12]. Subsequently, a 13-item short version was developed in two versions: one for chronic conditions and one for non-chronic conditions, with minor distinctions in the wording. The short form of the PAM has the same psychometric properties as the longer version and was found to be as valid and reliable [11].

Since the development of the PAM, a growing amount of research, mainly from the US, has shown that increased scores on the PAM are associated to a broad range of health-related outcomes. In the original validation studies by Hibbard and colleagues, higher activation scores corresponded with better health, lower rates of health care utilization, higher engagement in disease-specific self-management behaviors, a higher engagement in consumerist health behaviors, and preventive behaviors [9,11]. Further studies suggested that patient activation is strongly related to health behaviors e.g. engagement in regular exercise, managing stress, paying attention to diet and taking diabetes medications, less smoking behavior, or increased probability of having a breast cancer screen [12-15].

So far, the original English version of the PAM13 has been translated into Danish [16], Dutch [17], and Norwegian [18]. Furthermore, the concept has been applied to other conditions [19].

The aims of the present study were:

● to translate and adapt the American short form of the Patient Activation Measure (PAM13) into a German version.

● to establish the psychometric properties of the German version of the PAM13 in an multimorbid population with various chronic conditions.

## Methods

The PAM13 version for chronic conditions is the version most commonly used and will be described in further detail and analyzed in this study.

### Translation and adaptation

The original American version of the PAM13 was translated into German by a German native speaker with excellent knowledge of English and a background in psychology and health services research. Back-translation was done by a native English speaker in order to control for the original meaning of the words to be maintained. Any discrepancies in the translation process were discussed with both translators and two further team members of the research project who are experienced in translation processes of instruments and the field of research.

### Participants

The German version of the PAM13 was sent to N = 9.075 participants enrolled in a randomized controlled study set up to compare a telephone based health coaching with usual care (German Clinical Trials Register, DRKS00000584). The participants were all German health fund members and older than 18 years with a diagnosis of at least one chronic disease based on the routine data of a health fund. Their risk of rehospitalization within the next year was predicted higher than 50% based on health care costs, ICD-diagnoses, age and gender. The German version of the PAM13 was sent out as a paper-pencil version as part of a larger survey on patient reported outcomes to intervention and control group participants. Participants received a follow-up survey 12, 24 and 36 months after the first questionnaire. For this validation study only data from the first measure point (baseline) were used, before any intervention was administered.

### Ethics

Ethic approval was attained from the Ethics Committee of the State Chamber of Physicians in Hamburg (Germany). All participants gave informed content.

### Measures

To assess the demographic background of the patients, questions regarding gender, age, education, current work status and types of chronic disease were included in the questionnaire. Participants’ self-reported-health was determined with the first item of the short-form health survey (SF-12) [20]. Patient activation was measured with the German version of the PAM13 (see Table 1 for the content of the original version of the PAM13; see Additional file 1 for the German version of the PAM13). Each of the 13 items can be answered with one of four possible response options, which are “disagree strongly” (1), “disagree” (2), “agree” (3), “agree strongly” (4). A fifth response option “not applicable” (N/A) was given in the American version for all items. In the German version only item 4 had a fifth response option, namely “I do not take medications”. The authors considered this wording better understandable. The remaining items are general statements which should be applicable to all participants. Based on responses to the 13-item measure, the score is calculated by adding up the raw scores and mapping up the value onto a scale of 0–100 indicating strength of agreement with the 13 items. The final score can be assigned to one of the four levels of activation. A higher score indicates that the patient is likely to participate more actively in health care processes and takes more responsibility for his or her health.

**Table 1:** **The original American version of the Patient Activation Measure (13-item-form)**[11]

**Item**	
1	When all is said and done, I am the person who is responsible for managing my health condition
2	Taking an active role in my own health care is the most important factor in determining my health and ability to function
3	I am confident that I can take actions that will help prevent or minimize some symptoms or problems associated with my health condition
4	I know what each of my prescribed medications do
5	I am confident that I can tell when I need to go get medical care and when I can handle a health problem myself
6	I am confident I can tell my health care provider concerns I have even when he or she does not ask
7	I am confident that I can follow through on medical treatments I need to do at home
8	I understand the nature and causes of my health condition(s)
9	I know the different medical treatment options available for my health condition
10	I have been able to maintain the lifestyle changes for my health that I have made
11	I know how to prevent further problems with my health condition
12	I am confident I can figure out solutions when new situations or problems arise with my health condition
13	I am confident that I can maintain lifestyle changes like diet and exercise even during times of stress

### Statistical methods and analysis

For a better comparability of results of the German PAM13 to the American, Dutch, and Danish versions, the psychometric elements of the PAM13 were assessed in two phases.

In a first step, the data were analyzed for missing data. Participants with more than 30% missing values in the PAM13 were excluded from further analyses. For participants with missing data less than 30%, missing values were imputed using expectation-maximization procedure within the statistical software PASW Statistics (Version 18.0). Furthermore, data were described with respect to the percentage of missing responses, frequencies for the response options, mean, standard deviation and median for each item.

The internal consistency of the instrument was examined using Cronbach’s α for each item, as well as the inter-item and the item-rest correlation (Pearson’s r). An α of 0.70 was defined as the lowest acceptable value [21]. For the item-rest correlation correlations r ≥ 0.10 were considered as weak and correlations of r ≥ 0.30 as moderate and of r ≥ 0.50 as strong [22]. In the second step of the examination of the psychometric elements of the German version of the PAM13 the Andrich’s Rasch Rating Scale Model was used [23,24]. For this purpose the data was imported to the free statistical program R (R version 2.15.2, The R Foundation for Statistical Computing); the package eRm was used for the analysis of the Rating Scale Model [25]. Item and person parameters, model-fit-statistics, and differential item functioning (DIF) were investigated.

The Rasch model calibrates the difficulty of the items in relation to the response probabilities of a person, presuming that a single construct drives item responses [26]. This implies that the response of a person to an item informs on the person’s level on this construct. The probability of a person for a positive response of an item is in the Rasch model a logistic function of the item difficulty and person ability [27]. This logistic function is an interval scale where the midpoint is 0. The items are ordered on the scale respectively to their difficulty level; items on the top of the scale have lower probability that a person scores high on it (“more difficult” items) and on the bottom of the scale items have higher probability to be responded to (“less difficult” items) [23]. The item difficulty calibrations (Rasch Andrich thresholds) of the Rasch model contain information of the precision and monotonicity of the response category used [24]. If estimates are disordered, this indicates that the categories do not sufficiently reflect the intervals of the latent variable and response categories were not used. The model posits that thresholds that increase by at least 1.4 logits reflect sufficient distinction of response options and measurement model fit to reflect the sufficient distinction between the response options and measurement model fit. More than 5.0 logits indicate that intervals in the variable are too large [23,28].

Unidimensionality and local independence of the 13 items were examined as a requirement for the Rasch model [23]. This was addressed by using a Principal Components Analysis (PCA). The aim of the PCA of residuals is to extract one common factor that explains most of the variance in the analyzed items. The condition was considered as violated if other factors besides the common factor appear to have eigenvalues greater than 3 [27].

To test whether the items fit the expected model, the mean square information-weighted statistic (infit) and the outlier-sensitive statistic (outfit), were computed. The infit statistics are more sensitive to irregular response patterns according to the person’s ability level, whereas the outfit statistics informs about the degree of the item fit [23]. A good fit with the Rasch model indicate indices between 0.6 and 1.4 [16].

Equivalent to the Danish version of the PAM13, DIF was assessed from the Rasch model using the Andersen’s Likelihood Ratio Test. There should be no differences in the probability to endorse a certain item for subgroups [26,29]. Thus, DIF can be interpreted as a sign for the fairness of a test [16]. The DIF was assessed the Andersen’s Likelihood Ratio Test [25] for subgroups in sex, education, age and self-rated health.

## Results

### Participants

A total of N = 4.309 responded to the questionnaire, this is a response rate of 47.5%. Participants with more than 30% missing data were excluded from further analysis. After exclusion the total sample was N = 4.018. Participants were between 19 and 87 years old, the mean age was 67 years. Gender was distributed almost evenly with 54.8% female and 45.2% male participants. Regarding the ICD-10 diagnosis, 66.4% of the participants had arthrosis, 67.3% a heart insufficiency or coronary artery disease, 37.7% a lung disease, 41.9% cancer, 40.1% depression, 46.3% diabetes, 90.2% hypertension, 43.1% an anxiety disorder, and 38.5% adipositas. Most of the participants had more than one diagnosis. Education of the participants (N = 3.231) was divided into three levels similar to the validation studies from the USA, Netherlands and Denmark. Low educational level (no apprenticeship = 8.88%, other apprenticeship = 6.50%), middle educational level (apprenticeship = 64.9%), high educational level (technical college = 14.3%; university = 6.3%). Results are presented in Table 2.

**Table 2:** Demographic data and mean activation scores of the PAM13 for the German sample

	**N**	**%**	**PAM 13 Score**
**Sample**	4018	100	67.1
***Gender***			
**Male**	1796	44.7	68.1
**Female**	2180	54.3	66.9
***Age Groups***			
**−44**	116	2.9	64.4
**45-54**	272	6.8	64.5
**55-64**	839	20.9	66.6
**65-74**	2093	52.1	67.8
**75-84**	646	16.1	67.1
**+85**	10		53.1
***Education***			
**Low**	2353	58.6	66.5
**Middle**	673	16.7	67.2
**High**	205	5.1	70.7
***Self-rated Health***			
**Poor**	469	11.7	58.7
**Fair**	2057	51.2	65.0
**Good**	1324	33.0	72.2
**Very Good**	59	1.5	82.0
**Excellent**	11	0.27	85.1
***Diagnosis***			
**Heart problem**	2704	67.3	66.9
**Arthrosis**	2667	66.4	66.8
**Depression**	1616	40.1	64.9
**Lung Disease**	1514	37.7	66.1
**Cancer**	1684	41.9	67.5
**Diabetes**	1859	46.3	67.3
**Hypertension**	3626	90.3	67.1
**Anxiety**	1731	43.1	65.9
**Adipositas**	1548	38.5	66.4

Furthermore, Table 2 shows the mean activation scores of the German sample. In this sample men (68.1) have slightly higher activation score than women (66.9). In the distinction by age groups, the group with an age 65 from 74 years had the highest activation score (67.8) and the group of the over 85 years old the lowest (53.1). Regarding self-rated health patient activation is found being highest for the group with excellent health (85.1) and lowest for the group with poor health (58.7). When differentiating for diagnosis, the highest activation scores were found for the group of patients with cancer (67.5) and the lowest with depression (64.9).

### Psychometric properties

For the data description, the percentage of missing responses, the frequencies for the response options mean, standard deviation (SD) and median for each item were assessed (Table 3).

**Table 3:** Data description of the German version of the PAM13

**Item**	N*	Missing values*	Strongly disagree*	Disagree*	Agree*	Strongly agree*	Mean*	SD*	Inter-rest-correlation*
**1**	3970	1.2%	137	479	2359	1043	3.07	0.71	0.46
			(3.4%)	(11.9%)	(58.7%)	(26.0%)			
**2**	3961	1.4%	38	160	2693	1127	3.22	0.56	0.51
			(0.9%)	(4.0%)	(67.0%)	(28.0%)			
**3**	3958	1.5%	127	629	2531	731	2.96	0.68	0.53
			(3.7%)	(15.7%)	(63%)	(18.2%)			
**4**	3422	14.8%	38	265	2534	1079	3.19	0.59	0.51
			(0.9%)	(6.6%)	(63.1%)	(26.8%)			
**5**	3977	1.0%	117	652	2482	767	2.97	0.68	0.51
			(2.9%)	(16.2%)	(61.8%)	(19.1%)			
**6**	4003	0.4%	41	291	2493	1193	3.20	0.61	0.53
			(1.0%)	(7.2%)	(62.0%)	(29.7%)			
**7**	3988	0.7%	30	275	2612	1101	3.19	0.58	0.59
			(0.7%)	(6.8%)	(65.0%)	(27.4%)			
**8**	3987	0.8%	47	242	2391	1238	3.12	0.63	0.60
			(1.2%)	(8.5%)	(59.5%)	(30.8%)			
**9**	3983	0.9%	87	821	2380	730	2.93	0.68	0.63
			(2.2%)	(20.4%)	(59.2%)	(18.2%)			
**10**	3970	1.2%	50	489	2820	730	3.02	0.58	0.63
			(1.2%)	(12.2%)	(70.2%)	(16.4%)			
**11**	3971	1.2%	158	1120	2208	532	2.78	0.72	0.65
			(3.9%)	(27.9%)	(55.0%)	(13.2%)			
**12**	3862	3.9%	189	1123	2348	358	2.72	0.69	0.55
			(4.7%)	(27.9%)	(58.4%)	(8.9%)			
**13**	3869	3.7%	152	1006	2457	403	2.77	0.67	0.48
			(3.8%)	(25.0%)	(61.1%)	(10.0%)			

*based on the imputed data sample N = 4.018 and for item 4 N = 3.913 (n = 105 (2.6%) answered item 4 with “I do not take medicine”).

In general, acceptance of the items was high with percentages of missings per item ranging between 0.4% (item 6) and 3.9% (item 12). Only item 4 had 14.8% of missing answers.

The frequencies of the response categories showed little use of the category “strongly disagree” (from 0.7% to 4.7%) and moderate frequency for “disagree” (from 4.0% to 27.9%). The category “agree” was used with the highest frequency (from 55.0% to 70.2%) and the category “strongly agree” was moderately used (from 8.9% to 29.7%). The category “not applicable” for item four was used by 2.6% of the sample.

The median score was 3 for all items, the mean scores varied from 2.72 (item 12) to 3.20 (item 6). The last three items of the scale have a lower mean score in comparison to the items at the beginning of the questionnaire. The items did not constantly follow the sequence from higher scores to lower scores, which were presented for the original American scale. Standard deviations varied 0.56 (item 2) to 0.72 (item 11).

Regarding internal consistency, a Cronbach’s α of 0.88 was found for the sum scale. The item-rest-correlation, indicating the correlations between items and the sum of the other items were moderate to strong and ranged from 0.46 to 0.63 supporting the assumption of unidimensionality of the construct.

### Rasch analysis

One of the criteria to conduct adequate functioning of rating scale categories is that the category frequencies are fairly similarly distributed across items [27]. This criterion was not fulfilled as the response option “strongly disagree” was hardly used by the participants. Therefore, a new data set was created combining the categories “strongly disagree” and “disagree” into one response category. Further analyses were computed with this data set with only three response options. (Results for the data set including four response options can be found in the Additional file 2: Table S1).

#### Test of unidimensionality

The PCA for the testing of unidimensionality as a requirement for the Rasch model, could be confirmed. A total of 40.9% of the variance was explained by one factor with an eigenvalue of 5.3.

#### Rating scale model

The item statistics ranged from 0.68 to 1.03 for the infit (infit MSQ) and from 0.65 to 1.22 for the outfit (outfit MSQ) all indicating a good fit of the Rasch model. The thresholds increase by at least 1.4 logits showing sufficient distinction between the response options and measurement model fit. None of the thresholds exceeds the limit of 5.0 logits (Table 4). The item difficulty was presented by the location parameters of the items. Results show that the original order of difficulties found for the American version could not be confirmed for the German PAM13 (Table 4). For example, item 1 with a location parameter of 1.74 was more difficult for the German sample than item 8 with a location parameter of 1.27. Items 4 and 7 could be located at the same difficulty levels as the corresponding items of the American version. Consistent with the original version, items 11, 12 and 13 showed the highest difficulties.

**Table 4:** Item statistics

**Item**	**Location parameter**	**Threshold 1**	**Threshold 2**	**SE**	**Outfit MSQ**	**Infit MSQ**
**2**	1.17	−0.86	3.21	0.03	0.85	0.83
**6**	1.25	−0.78	3.29	0.03	0.94	0.94
**8**	1.27	−0.76	3.30	0.03	0.84	0.88
**4**	1.33	−0.70	3.37	0.03	0.95	0.92
**7**	1.33	−0.71	3.36	0.03	0.74	0.77
**1**	1.74	−0.29	3.78	0.03	1.22	0.94
**10**	2.09	0.06	4.13	0.03	0.65	0.68
**5**	2.23	0.19	4.26	0.03	1.03	1.03
**3**	2.26	0.22	4.29	0.03	1.00	1.01
**9**	2.42	0.39	4.46	0.03	0.88	0.90
**11**	3.04	1.00	5.07	0.03	0.87	0.88
**13**	3.05	1.01	5.09	0.03	1.05	1.03
**12**	3.26	1.23	5.30	0.03	0.93	0.93

The items are ranked according to their difficulty (location parameter). Information on thresholds, standard error (SE), and fit statistics (outfit and infit MSQ).

**Figure 1:**
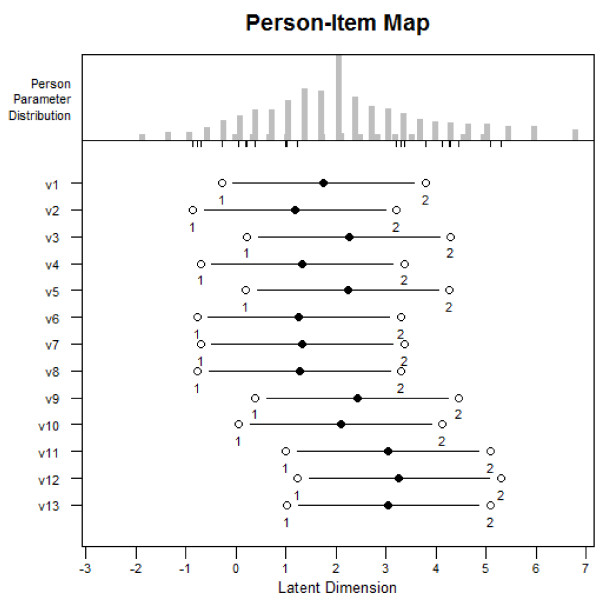
Person item-map.

The Rasch person-item map (Figure 1) displays the person parameter distribution on the latent dimension and the item difficulties. The black dots show the mean item-difficulty and white dots reflect the ranges of thresholds, as presented in Table 4. Persons on the left side of the scale report being less activated than persons on the right side of the scale.

#### Differential item functioning

The DIF test for sex showed statistically significant differences for women and men (LR-value: 122.61, df = 13, p = 0.001). Differences were most prominent for item 4 and 5 located on the second level “confidence and knowledge to take action”, which were slightly more difficult to endorse for men than for women. Item 1, located on the first level of the construct “believes active role is important”, was slightly more difficult for women.

DIF according to education was tested by comparing the low education group with the collapsed middle and high education group. For education no DIF in subgroups was found (LR-value: 14.74, df = 13, p = 0.324).

Furthermore, the DIF was tested for subgroups in age. The sample was divided into two groups (younger than 44 years to 64 years and 65 years to older than 85). The test revealed significant differences (LR-value: 184.02, df = 13, p < 0.001). Item 2 belonging to the first level of patient activation, item 3 located on the second level of the construct which is “confidence and knowledge to take action”, item 11 located on the third level “taking action” and item 12 belonging to the fourth level “staying in course under stress” were more difficult to endorse for the older age group. Item 13, also located on the fourth level was slightly easier to endorse for the older age group than for the younger age group.

Finally, the DIF was tested for subgroups in self-rated health. Two subgroups were formed with the first group consisting of persons who rated there health as excellent, very good or good and a second group including persons with a fair or poor rating of their health. The results showed evidence of DIF (LR-value: 268.47, df = 13, p < 0.001). Differences were found for the items 2 (level 1), the items 6, 7 (level 2), and 9, 10 (level 3). These items were easier for the subgroup in which self-rated-health was fair or poor.

DIF was especially found for the second cut off value between the response options “agree” and “totally agree”.

The graphical output for the DIF testing can be found in the Additional file 3: (Figure S1 to Figure S4).

## Discussion

The German version of the PAM13 was tested in a multimorbid population with various chronic conditions. The German version showed good internal consistency (Cronbach’s α = 0.88) and inter-rest correlations were moderate to strong. The findings for the internal consistency are comparable to the Danish and the Dutch version of the instrument. For the American version, no Cronbach’s α was published. The analysis of the frequencies of the item response options revealed that the response category “strongly disagree” was very rarely used and the category “disagree” was frequented very seldom as well. The response category “agree” was chosen the most. The irregular use of response options can be interpreted as a lack of fit of the response scale with the study sample.

The Rasch analysis of the items showed that the original difficulty ranking order of the items could not be confirmed. The differences in the item order indicate that the German population found other items easier or more difficult to respond to than the American population. E.g. item 1 and 3 were rated as more difficult in the German sample than item 6 and item 8. Consistent with the American version, items 11, 12 and 13 were the most difficult items of the scale, yet the order differed slightly in the German version (11, 13, 12). This could be due to specifics of this sample (age, multimorbidity, various chronic diseases) or cultural differences between the American and the German samples. Since the Dutch and Danish validation studies could not confirm the original item order either, differences in the European and American understanding of health system may be the cause of these results. Comparing the Dutch, Danish and German item order, few similarities can be found, the Danish and Dutch item orders being more similar to the original American item order. E.g. item 7 was also less difficult for the Danish sample and item 5 more difficult in comparison to the original order of the American version. Item 3 was found as very difficult in the German sample but was in the “correct” order in the Danish validation. It is important to note that the Dutch validation study did not use Rasch modeling. The Norwegian validation study of the PAM13 is not available in English and therefore no comparisons were made with this study.

Comparing the activation scores of subgroups in the German sample, self-rated health is the most distinguishing variable, which is similar to the results of the Dutch, Danish and American validation studies. However, as all studies use a cross-sectional design they cannot answer the question of cause and effect. The activation scores for the diagnosis subgroups differ only slightly with the lowest scores attained by the depression and anxiety group. Considering that patients with these diagnoses generally have low self-esteem, are caught up in negative thoughts and emotions, and usually express low levels of action and self-management, this result is not surprising, but scores in this subgroup are still considerably high [10,17]. When testing for DIF, several differences for the difficulty of certain items were revealed in the subgroups of sex, age, and self-rated health, but these differences can be seen as rather small.

Referring to the current state of research little is known about the cross-cultural validity of the PAM13. A Danish, Dutch, and Norwegian translation have been analyzed so far and translations into further other languages and testing of the psychometric properties among different cultures remains outstanding [11]. Furthermore, comparison of the existing validation studies is limited because of different cultural contexts, sample sizes, age groups and diagnoses.

### Strengths and limitations

A strength of this study is the sample size of 4.018 persons, which is notably bigger than those of the other validation studies. Moreover, the sample presents a heterogeneous group of patients with various chronic conditions similar to the original American validation study. It could be assumed that a heterogeneous sample scores on all levels of activation [16], but this could not be confirmed in the present study, as ceiling effects were very high. Another strength of this study is the high responsiveness of the items with only 0.4% to 3.9% missings per item. An exception was item 14 with 14.8% missing values. The reason for this deviating result can only presumed by the authors. Since this item is the only one which clearly refers to medication, it is possible that some participants are not firm on their medication and did not feel comfortable to respond to this item.

As limitations of the study the following points are discussed. First, the comparability of the German translation to other validation studies might be limited due to the fact, that the response options were changed by omitting the option “not applicable”. The authors were of the opinion that besides item 4 all statements should be applicable and the response option “not applicable” might cause a loss of information. Nevertheless, this could be reconsidered in further validation studies.

Second, after a first analysis the response categories “strongly disagree” and “disagree” were combined into one response category. Again this might be limit the comparability of this study, but adequate functioning of rating scale categories requires that the category frequencies are fairly similarly distributed across items [27]. The authors still did the calculation on the data with four response options and provided these results in the Additional file 2: Table S1).

Third, the overall response rate of the study was low with 47.5%. When comparing non-responders to the responders it was found that they differed in age with the responders being older (mean = 67 years) than the non-responders (mean = 65.5 years). There were slightly more women than men with the non-responders but this might due to the fact, that slightly more women were included into the study in the first place. There was no other information e.g. about the health of the non-responders as this information was only accessible when the survey was completed. It is possible that especially non-responders have lower PAM scores, the responders of this study participants scored very high on the PAM and low levels of the PAM construct were rarely used.

As a final limitation it should be mentioned that during the translation process, comprehensibility and equivalence were not discussed with groups of patients as it was done in the Danish validation study. Only researchers were involved in our translation process.

#### Implications for practice and further research

With regard to the current problems the health care system has to face, e.g. a growing number of elderly people and increasing numbers of people living with a chronic disease [2], enhancing patients’ involvement in their healthcare is one option to improve the situation. Especially older patients with various diseases have difficulties to get involved in their treatment. Measuring the level of a person’s activation can help to improve the success of interventions by tailoring them according to the individual’s level of activation and the corresponding readiness to be engaged. To that end, the PAM13 can be a useful instrument. However, different aspects are recommended to address in further research before using the German version of the PAM13 in practice. First, longitudinal studies and interventions studies with the German version of the PAM13 are necessary to test for change over time and effects of patient activation. Intervention studies are mainly carried out in the United States so far. For example, Shively et al. [30] found a significant increase of the PAM13 scores for patients with chronic heart failure after participating in a self-management training for six months. First studies that are testing the instrument and its correlations with different outcome measures have been also done in the Netherlands. Rademakers and collegues [31] examined the effect of patient activation and health literacy on provider choice, and found that low levels on these constructs were negatively associated with active provider choice. Second, it would be recommended to test the German PAM13 with other samples, e.g. also non-clinical samples. Earlier American studies (e.g. [32,33]) found that the PAM13’s psychometric properties are also robust in samples with employees. Activation was found as directly related to the health status and job performance measures. Finally, the different item order found for the German version as well as the differences for subgroups in sex, age and self-rated health for the probability to endorse a certain item raise the question whether the Rating Scale Model is the best fitting model for this construct or whether e.g. a Partial Credit Model, which is less restricted, would show a better fit. Moreover, the high ceiling effects that leaded to the merging of the first and second response option were a problem in this validation study. Future research should test again whether the original model is applicable to the German population.

## Conclusion

Thus far, only a very few solely validation studies regarding the PAM13 were conducted in other countries than the USA. Most of the intervention and validation studies were done by the developers of PAM13 (Hibbard and colleagues) or by researchers who are associated with the team. With the validation of the German version of the PAM13, a further study investigating the construct is available. The results of the German validation study indicate that the reliability of the construct and model fits for the Rating Scale Model are good. Nevertheless, the original order of the items was not confirmed and the irregular use of the response options led to difficulties for the analyses and calls for more research and development on the construct. Before using the German version of the PAM13 in daily practice, further validation studies on the construct validity are highly recommended.

## Competing interests

The authors declare that they have no competing interests.

## Authors’ contributions

JD and MH conceived the study and sought funding. SD was responsible for the translation process and the data collection. JZ was responsible for the statistical analyses and interpretation of the data in correspondence with LK and AR. JZ wrote the first draft of the manuscript and did the revision. All authors contributed to subsequent drafts of the manuscript and have read and approved the final version.

## Pre-publication history

The pre-publication history for this paper can be accessed here:

http://www.biomedcentral.com/1471-2458/13/1027/prepub

## Supplementary Material

Additional file 1 Patient Activation Measure - 13 (PAM13) - German Version. Click here for file

Additional file 2 Item statistics for the data sample with four response options. Click here for file

Additional file 3 Figures of differential item functioning. Click here for file
