# Supplementary material for: Psychometric evaluation of the German version of the patient activation measure (PAM13)
Source: BMC Public Health. 2013 Oct 30;13:1027. doi: 10.1186/1471-2458-13-1027 (PMC4228438; doi:10.1186/1471-2458-13-1027)
Supplement: Additional file 1 — Patient Activation Measure - 13 (PAM13) - German Version. [file 1471-2458-13-1027-S1.docx]

**Patient Activation Measure (PAM13) - Deutsche Version**

| **Autor(inn)en** | Jördis M. Zill, Sarah Dwinger, Levente Kriston, Anja Rohenkohl, Martin Härter, Jörg Dirmaier |
| --- | --- |
| **Originalversion** | Kurzversion: Hibbard, J. H., Mahoney, E. R., Stockard, J., & Tusler, M. (2005). Development and testing of a short form of the patient activation measure. *Health Services Research,* 40:6 Part 1.  Orginalversion: Hibbard, J. H., Stockard, J., Mahoney, E. R., & Tusler, M. (2004). Development of the Patient Activation Measure (PAM): conceptualizing and measuring activation in patients and consumers. *Health Services Research, 39.*  Übersetzungen: Rademakers, J., Nijman, J., van der Hoek, L., Heijmans, M., & Rijken, M. (2012). Measuring patient activation in the Netherlands: translation and validation of the American short form Patient Activation Measure (PAM13). *BMC Public Health, 12*(1), 577.  Maindal, H. T., Sokolowski, I., & Vedsted, P. (2009). Translation, adaptation and validation of the American short form Patient Activation Measure (PAM13) in a Danish version. *BMC Public Health, 9*, 209. |
| **Anwendungsbereiche** | Der Patient Activation Measure (PAM13) wurde zur Anwendung in der klinischen Praxis und der Forschung entwickelt. Es werden das Wissen, die Fähigkeiten und die Zuversicht Gesundheitsbeschwerden selbst zu bewältigen, erfasst. |
| **Zielsetzungen und Kurzbeschreibung** | Der Patient Activation Measure-13 (PAM13) wurde theoriegeleitet entwickelt. Das Instrument misst die Aktivierung von Patienten im Umgang mit ihrer Gesundheit. Dabei können die Patienten vier Stufen der Aktivierung zugeordnet werden : (1) Überzeugung, dass die aktive (Patienten-) Rolle wichtig ist (2) Zuversicht and Wissen um aktiv zu werden (3) Aktiv sein (4) Aktiv bleiben, auch unter Stress. |
| **Art des Verfahrens** | *Selbstbeurteilungsverfahren* |
| **Aufbau** | Die Kurzfassung des Instruments zur Patientenaktivierung besteht aus 13 Items, welche den vier Aktivierungsstufen zugeordnet sind. Die Items werden auf einer 4-stufigen Likert Skala erfasst (1 = stimme überhaupt nicht zu, 2 = stimme nicht zu, 3 = stimme zu, 4 = stimme voll und ganz zu). Für das 4. Item gibt es zusätzlich die Antwortoption «Ich nehme keine Medikamente ein». |
| **Statistische Auswertung/Scoring** | Die Auswertung erfolgt über die Addition der Rohwerte (Range 13-52). Es wird empfohlen, fehlende Werte durch den Mittelwert zu ersetzen. Bei weniger als neun vollständig ausgefüllten Antworten (ca. 30%) sollte auf eine Auswertung verzichtet werden. Zur Standardisierung des Rohgesamtwertes wird eine Transformation auf eine 0-100 Skala empfohlen (100 *(Summe - 13) / (52 - 13)). |
| **Interpretation** | Höhere Summenwerte weisen auf eine höhere Patientenaktivierung hin. Der finale Wert kann den 4 Stufen der Aktivierung zugeordnet werden. Ein Patient mit einem hohen Wert kann sich besser aktiv in den Behandlungsprozess einbringen und mehr Verantwortung für seine Gesundheit übernehmen. |
| **Gütekriterien** | Objektivität  Die Durchführungs-, Auswertungs- und Interpretationsobjektivität sind durch die standardisierte Darbietung und Auswertungsinstruktionen gegeben.  Reliabilität  Die interne Konsistenz kann als sehr gut befunden werden (Cronbach’s α = 0.88). Die Inter-Rest-Korrelationen der Items variierten zwischen moderat und hoch (0.46 bis 0.63) und weisen damit auf die Unidimensionaltität des Konstruktes hin. Die in der amerikanischen Version des Instruments gefundene Reihenfolge der Items konnte in der deutschen Adaption nicht gefunden werden.  Retest-, Paralleltest- und Testhalbierungs-Reliabilität liegen nicht vor.  Validität  Konstruktvalidität: alle Items laden auf einem Faktor (40.9% Varianzaufklärung).  Angaben zur konvergenten Validität, Kriteriumsvalidität und Veränderungssensitivität liegen nicht vor.  Akzeptanz:  Beantwortungsquoten von über 95% deuten auf eine hohe Akzeptanz hin. Nur für Item vier war die Akzeptanz mit 85% niedriger. |
| **Vergleichswerte/Normen** | Bisher liegen keine Normwerte für bestimmte Erkrankungsbilder vor. |
| **Kurzversionen** | Bei der dargestellten Version handelt es sich um die revidierte Kurzform des Instrumentes mit 13 Items. Die Originalversion beinhaltet 22 Items. |
| **Kontakt** | Jördis M. Zill, Dipl.-Psych.   Institut und Poliklinik für Medizinische Psychologie Zentrum für Psychosoziale Medizin Universitätsklinikum Hamburg-Eppendorf Martinistr. 52 (Haus W26) 20246 Hamburg Tel: +49 (040) 7410 58649 Email: [j.zill@uke.de](mailto:j.zill@uke.de) |

| 1. **Im folgenden Abschnitt möchten wir von Ihnen gerne erfahren, was Sie über Ihre Erkrankung wissen und wie sicher Sie sich in Bezug auf Ihren Umgang mit Ihrer Erkrankung fühlen.**   **Bitte kreuzen Sie für jede der folgenden Aussagen ein Kästchen an, das für Sie persönlich am besten beschreibt, wie sehr Sie der Aussage zustimmen oder nicht zustimmen. Es gibt keine richtigen oder falschen Antworten. Ihre Antworten sollten Ihre persönliche Meinung widerspiegeln, unabhängig davon, was Ihr Arzt gerne von Ihnen hören würde.** |
| --- |

|  | stimme überhaupt nicht zu | | stimme nicht zu | stimme zu | stimme voll und ganz zu |
| --- | --- | --- | --- | --- | --- |
| Letzten Endes bin ich selbst dafür verantwortlich, mich um meine  Erkrankung zu kümmern. | ❑ | | ❑ | ❑ | ❑ |
| Um meine Gesundheit und meine Leistungsfähigkeit zu beeinflussen, ist es am  wichtigsten, eine aktive Rolle im Rahmen meiner Behandlung einzunehmen. | ❑ | | ❑ | ❑ | ❑ |
| Ich bin davon überzeugt, dass ich Maßnahmen ergreifen kann, die helfen, Symptome und Probleme meiner Erkrankung zu verhindern oder zu verringern. | ❑ | | ❑ | ❑ | ❑ |
| Ich weiß, was jedes der mir verschriebenen Medikamente bewirken soll. | ❑ | ich nehme keine Medikamente ein  **(weiter bei nächster Frage)** | | | |
|  | ❑ | | ❑ | ❑ | ❑ |
| Ich bin davon überzeugt, dass ich beurteilen kann, wann ich eine medizinische Behandlung benötige und wann ich ein Gesundheitsproblem selbst bewältigen kann. | ❑ | | ❑ | ❑ | ❑ |
| Ich bin davon überzeugt, dass ich meinem Behandler meine Anliegen mitteilen kann, auch wenn er nicht danach fragt. | ❑ | | ❑ | ❑ | ❑ |
| Ich bin davon überzeugt, dass ich medizinische Behandlungen, die ich zu Hause durchführen muss, auch umsetzen kann. | ❑ | | ❑ | ❑ | ❑ |
| Ich weiß über meine Erkrankung und ihre Ursachen Bescheid. | ❑ | | ❑ | ❑ | ❑ |
| Ich kenne die verschiedenen Behandlungsmöglichkeiten für meine Erkrankung. | ❑ | | ❑ | ❑ | ❑ |
| Ich bin in der Lage, die Veränderungen meiner Lebensgewohnheiten aufrechtzuerhalten, die ich aufgrund meiner Erkrankung begonnen habe. | ❑ | | ❑ | ❑ | ❑ |
| Ich weiß, wie ich weitere Probleme mit meiner Erkrankung verhindern kann. | ❑ | | ❑ | ❑ | ❑ |
| Ich bin davon überzeugt, dass ich für neu auftretende Situationen oder Probleme mit meiner Erkrankung Lösungen finden kann. | ❑ | | ❑ | ❑ | ❑ |
| Ich bin davon überzeugt, dass ich Veränderungen meiner Lebensgewohnheiten, wie eine gesunde Ernährung und Sport, auch in stressigen Zeiten beibehalten kann. | ❑ | | ❑ | ❑ | ❑ |
