# Supplementary material for: Psychometric evaluation of the German version of the patient activation measure (PAM13)
Source: BMC Public Health. 2013 Oct 30;13:1027. doi: 10.1186/1471-2458-13-1027 (PMC4228438; doi:10.1186/1471-2458-13-1027)
Supplement: Additional file 2 — Item statistics for the data sample with four response options. [file 1471-2458-13-1027-S2.docx]

**Additional file 2: Item statistics for the data sample with four response options**

**Table A1: Item statistics**

| Item | Location Parameter | Threshold 1 | Threshold 2 | Threshold 3 | SE | Outfit MSQ | Infit MSQ |
| --- | --- | --- | --- | --- | --- | --- | --- |
| 2 | 1.46 | -0.79 | 0.59 | 4.57 | 0.03 | 0.86 | 0.86 |
| 6 | 1.53 | -0.71 | 0.66 | 4.65 | 0.03 | 0.96 | 0.98 |
| 8 | 1.55 | -0.69 | 0.68 | 4.66 | 0.03 | 0.87 | 0.93 |
| 7 | 1.59 | -0.65 | 0.72 | 4.70 | 0.03 | 0.75 | 0.79 |
| 4 | 1.60 | -0.64 | 0.73 | 4.72 | 0.03 | 0.95 | 0.94 |
| 1 | 2.08 | -0.17 | 1.20 | 5.19 | 0.03 | 1.33 | 1.35 |
| 10 | 2.29 | 0.04 | 1.42 | 5.40 | 0.03 | 0.62 | 0.65 |
| 5 | 2.46 | 0.22 | 1.59 | 5.58 | 0.03 | 1.06 | 1.06 |
| 3 | 2.49 | 0.25 | 1.62 | 5.60 | 0.03 | 1.01 | 1.01 |
| 9 | 2.59 | 0.35 | 1.72 | 5.71 | 0.03 | 0.83 | 0.83 |
| 11 | 3.11 | 0.87 | 2.24 | 6.23 | 0.03 | 0.81 | 0.77 |
| 13 | 3.12 | 0.87 | 2.25 | 6.23 | 0.03 | 1.00 | 0.94 |
| 12 | 3.29 | 1.05 | 2.42 | 6.41 | 0.03 | 0.88 | 0.83 |

Thirteen items are ordered according to their positions in the difficulty hierarchy (location parameter). Information on thresholds, standard error (SE) and fit statistics (outfit and infit MSQ)
