# Supplementary material for: Psychometric evaluation of the German version of the patient activation measure (PAM13)
Source: BMC Public Health. 2013 Oct 30;13:1027. doi: 10.1186/1471-2458-13-1027 (PMC4228438; doi:10.1186/1471-2458-13-1027)

**Additional file 3: Figures of differential item functioning (DIF)**

Items above the diagonal are more difficult to endorse for the group located on the y-ordinate and vice versa.

**Figure A1:** Graphical output for DIF in sex. Group 1 = female (N = 2180) vs. group 2 = male (N = 1.796).


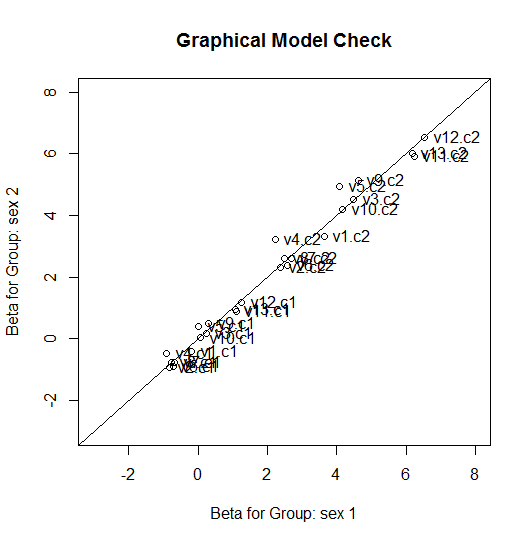


**Figure A2:** Graphical output for DIF in education. Group 1 = low educational level (N = 2.353) vs. group 3 = middle and high educational level (N = 878).


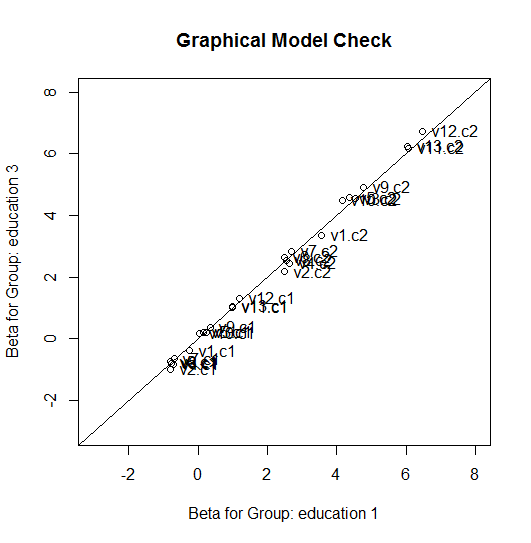


**Figure A3:** Graphical output for DIF in age. Group 2 = younger than 44 years to 64 years (N = 1.227) vs. group 3 = 65 years to older than 85 (N = 2.749).


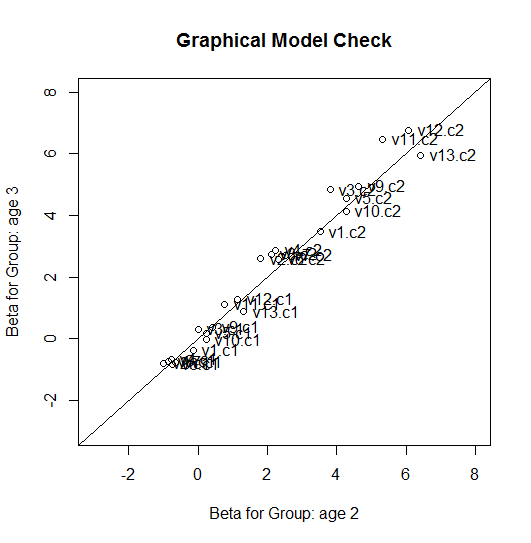


**Figure A4:** Graphical output for DIF in self-rated-health. Group 1 = excellent, very good, good (N = 2.526) vs. group 2 = fair, poor (N = 1.394).


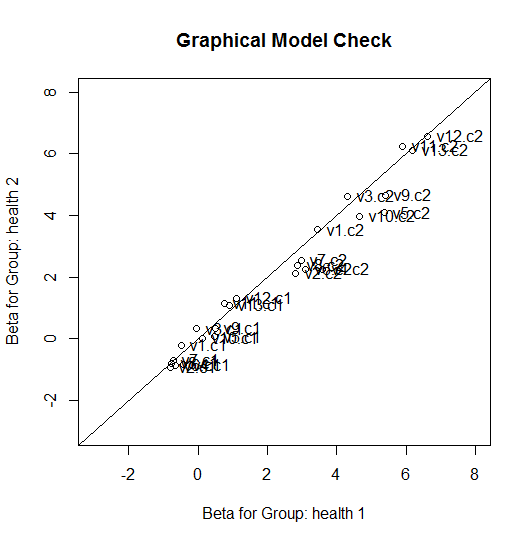

Supplement: Additional file 3 — Figures of differential item functioning. [file 1471-2458-13-1027-S3.docx]
